# Pharmacological (or Synthetic) and Nutritional Agonists of PPAR-γ as Candidates for Cytokine Storm Modulation in COVID-19 Disease

**DOI:** 10.3390/molecules25092076

**Published:** 2020-04-29

**Authors:** Carmen Ciavarella, Ilenia Motta, Sabrina Valente, Gianandrea Pasquinelli

**Affiliations:** Laboratory of Clinical Pathology, Department of Experimental, Diagnostic and Specialty Medicine (DIMES), S. Orsola-Malpighi Hospital, University of Bologna, 40138 Bologna, Italy; carmen.ciavarella2@unibo.it (C.C.); ilenia.motta@studio.unibo.it (I.M.); gianandr.pasquinelli@unibo.it (G.P.)

**Keywords:** cytokine storm, PPAR-γ, metabolism, inflammation, PPAR-γ agonists, coronavirus infection

## Abstract

The cytokine storm is an abnormal production of inflammatory cytokines, due to the over-activation of the innate immune response. This mechanism has been recognized as a critical mediator of influenza-induced lung disease, and it could be pivotal for COVID-19 infections. Thus, an immunomodulatory approach targeting the over-production of cytokines could be proposed for viral aggressive pulmonary disease treatment. In this regard, the peroxisome proliferator-activated receptor (PPAR)-γ, a member of the PPAR transcription factor family, could represent a potential target. Beside the well-known regulatory role on lipid and glucose metabolism, PPAR-γ also represses the inflammatory process. Similarly, the PPAR-γ agonist thiazolidinediones (TZDs), like pioglitazone, are anti-inflammatory drugs with ameliorating effects on severe viral pneumonia. In addition to the pharmacological agonists, also nutritional ligands of PPAR-γ, like curcuma, lemongrass, and pomegranate, possess anti-inflammatory properties through PPAR-γ activation. Here, we review the main synthetic and nutritional PPAR-γ ligands, proposing a dual approach based on the strengthening of the immune system using pharmacological and dietary strategies as an attempt to prevent/treat cytokine storm in the case of coronavirus infection.

## 1. Introduction

The rapid diffusion of the disease and the high mortality rate of the coronavirus 2020 outbreak pose a major threat to world public health. At the time of writing, more than 2,400,000 (20 April 2020) coronavirus cases have been reported worldwide, approximately half of them affecting the USA, Italy, Spain, and China [1], and this number is expected to grow linearly. Moreover, the largest epidemiological study done by the Chinese Center for Disease Control and Prevention (CDC) showed that among the 44,672 confirmed cases, 4.7% were critical pneumonia cases with a fatality rate of 49% [2].

The histological picture of this pulmonary illness is characterized by prominent alveolar damage with eosinophilic exudates, hyaline membrane formation, mononuclear inflammatory cells, multinucleated giant cells, severe pneumocyte hyperplasia, and interstitial thickening [3]. Among the main mechanisms associated with the deterioration of the disease, one of the most important is the cytokine storm [4], an abnormal host inflammatory response previously seen in some viral threats, e.g., H1N1 or the H5N1 influenza viruses.

This cytokine storm is characterized by an aggressive pro-inflammatory response with inadequate control of the counteracting anti-inflammatory network [5] and has been directly correlated with tissue injury and an unfavorable prognosis in the case of severe influenza [6]. Furthermore, in the critical patients markedly higher levels of pro-inflammatory cytokines, including interferons (IFNs), tumor necrosis factors (TNFs), interleukins (ILs), and chemokines, have been detected in the blood [7]. This severe pro-inflammatory response has been also related to multi-organ dysfunction, a characteristic of irreversible disease [6]. In this context, there are currently no treatments of proven efficacy capable of reducing the progression of the disease from mild-moderate to severe-critical and to reverse the cytokine storm [8].

In the absence of a specific treatment for this novel virus, there is an urgent need to explore alternative solutions and targets to prevent and control the insidious inflammatory effects of the virus by modulating positively the host immune response. In this regard, peroxisome proliferator-activated receptors (PPARs) are a family of transcription factors that belong to the ligand-activated nuclear hormone receptors (NR) superfamily and recently emerged as critical regulators of inflammation. The PPAR family includes three subtypes that are encoded by distinct genes and are named PPAR-α, PPAR-β/δ, and PPAR-γ [9]. The three PPAR isoforms share a common structure, but manifest different tissue distribution, target genes, and functions. All of them primarily regulate the lipid and glucose metabolism and have additional regulatory roles on cell proliferation and differentiation, cancer, vascular homeostasis and atherosclerosis, the immune system, and inflammation, in in vitro and in vivo investigations [10].

Therefore, one interesting approach to counteract the cytokine storm could be the use of drugs or natural substances able to activate PPARs consistently.

PPAR-γ agonists (e.g., rosiglitazone and pioglitazone) are considered to be the most promising candidates to improve the clinical outcome of severe viral diseases [11]. These thiazolidinediones (TZDs) not only down-regulate the inflammatory response to viral pneumonia, but also promote the survival of influenza-infected mice [12].

In addition to synthetic compounds, natural PPAR-γ agonists contained in food could represent an aid to human health by acting as anti-inflammatory molecules, activating PPAR-γ and its ability to inhibit the expression of pro-inflammatory cytokines and to modulate the differentiation of immune cells towards anti-inflammatory phenotypes [13].

We have done an online search on PubMed and Web of Science using “PPAR-γ and/or viral disease”, “PPAR-γ and agonist/s”, “PPAR-γ and fruit and vegetables” as keywords.

Here we summarize the pharmacological and nutritional interventions that can favorably modulate the immune system through PPAR-γ stimulation. These synthetic and natural agents strengthen individual health, in an attempt to prevent cytokine storm in the case of coronavirus infection.

## 2. PPAR-γ: Structure, Tissue Expression and Function

### 2.1. Structure of PPAR-γ

**Figure 1:**
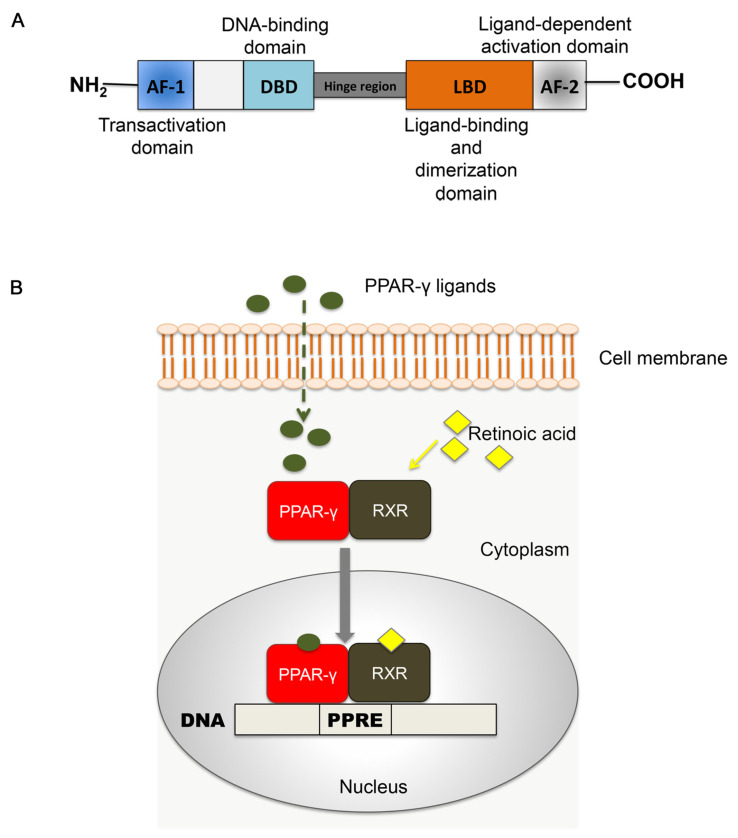
Peroxisome proliferator-activated receptors (PPARs). (**A**) Schematic representation of functional domains in PPAR. AF-1: domain of activation function 1; DBD: DNA-binding domain; LBD: ligand binding domain; AF-2: domain of activation function 2. (**B**) Mechanism of PPAR-RXR signaling. Upon ligand binding, the heterodimer PPAR: RXR translocates into the nucleus, where it binds to target DNA in correspondence with peroxisome proliferator hormone response elements (PPREs). This step initiates structural alterations essential to target gene modulation by PPARs.

PPAR-γ shares a common nuclear receptor structure with the other isoforms, composed of different functional domains. The NH_2_ terminal region contains a ligand-independent transactivation domain (AF-1) and a mitogen-activated protein phosphorylation site. The DNA-binding domain (DBD) is highly conserved and contains two zinc finger motifs responsible for receptor binding to DNA targets on the peroxisome proliferator hormone response elements (PPREs) [9]. A variable hinge region links the DBD domain to the ligand dimerization domain (LBD), at the COOH-terminus. The LBD is responsible for ligand specificity and is followed by the ligand-dependent activation domain (AF-2), crucial for the recruitment of PPAR coactivators essential to the gene transcription process [14]. A depiction of the PPAR structure is reported in Figure 1A.

The activation and functioning of PPARs require the heterodimerization with another nuclear receptor, the retinoid X receptor (RXR) [15]. The RXR family includes three different isoforms (RXR-α, -β, -γ) that are activated by endogenous 9-cis retinoic acid [16]. Upon ligand binding, the PPAR-RXR complex translocates into the nucleus, where it binds to the PPRE with the sequence AGGTCANAGGTCA on the promoter of target DNA. The association with the ligand induces conformational changes, corepressor dissociation and coactivator association, leading to ligand-induced trans-activation [17]. These structural and functional modifications initiate the modulating action of PPAR-γ-RXR on target gene transcription [16,18,19] (Figure 1B).

### 2.2. Tissue Expression

PPAR-γ exists in two main protein isoforms, as a consequence of alternative splicing at 5′ and alternative use of promoters. PPAR-γ2 is mainly expressed in white and brown adipose tissue, whereas PPAR-γ1 is widely present in almost all types of cells, including muscle, intestine, liver, kidney, and immune system [20]. The wide tissue distribution of PPAR-γ suggests its pleiotropic nature and that it supports a broad spectrum of activities.

### 2.3. Functions

Even if PPAR-γ is predominantly known for the regulation of glucose and lipid metabolism, this receptor possesses multiple functions, acting on different levels, such as intestine, brain, muscle, immune, and cardiovascular systems. 

PPAR-γ stimulates pre-adipocyte differentiation into adipocytes [21,22] and regulates the insulin sensitivity in peripheral tissues and the storage of fatty acids, through the modulation of genes involved in fatty acid release, transport, and storage into mature adipocytes. Further, PPAR-γ activates the transcription of glucose transporter type 4 (Glut4) and c-Cbl-associated protein (CAP), thus contributing to glucose metabolism [23].

In addition to the control of lipid and glucose metabolism, PPAR-γ also plays a role in a wide range of activities. The stimulation of PPAR-γ by docosahexaenoic acid (DHA) demonstrated a neuroprotective function on microglial cells, by reducing oxidative stress and improving mitochondrial functioning [24]. In vitro studies also propose PPAR-γ as a possible target for anti-cancer therapy, due to its anti-proliferative effects on cancer cell lines [25].

The expression of PPAR-γ has also been demonstrated on endothelial and vascular smooth muscle cells (SMCs), modulating oxidative stress, inflammation, and cell proliferation, which are crucial events during cardiovascular disease occurrence. Interestingly, PPAR-γ is expressed in macrophage-foam cells in human and murine atherosclerotic plaques [26,27], as well as in human and mouse monocyte and macrophages. In parallel, our research data showed that a low expression of PPAR-γ in mesenchymal stromal cells (MSCs) isolated from human abdominal aortic atherosclerotic plaques; moreover, we demonstrated that stimulation with inflammatory cytokines TNF-α/IL-1β reduced PPAR-γ expression in healthy vascular MSCs [28]. These findings support the inverse relationship between PPAR-γ and the inflammatory process and imply a regulatory ability of PPAR-γ on immune system and inflammatory circuits.

One of the mechanisms whereby PPAR-γ modulates inflammation is the interaction with the transcription factors belonging to the nuclear factor kappa light chain enhancer of activated B cells (NF-κB) family, which is the main regulator of immune response and inflammatory cascade. According to literature, PPAR-γ promotes NF-κB inactivation in different ways, by silencing pro-inflammatory gene inducers of NF-κB or alternatively by interacting with p65, essential to NF-κB signaling [29]. In addition, PPAR-γ induces the expression of antioxidant enzymes, reducing the production of reactive oxygen species (ROS), related to inflammatory reactions.

In addition to the expression in monocytes and macrophages, PPAR-γ was shown to inhibit monocyte differentiation into macrophages and to be crucial for macrophage M2 polarization [27,30], thus reducing the inflammatory cascade.

### 2.4. Synthetic Ligands of PPAR-γ

The synthetic agonists of PPAR-γ can be distinguished into two main groups: TZDs or glitazones and non-steroidal anti-inflammatory drugs (NSAIDs).

NSAIDs are anti-inflammatory, anti-pyretic, and analgesic drugs acting through the inhibition of cyclooxygenases (COX-1, COX-2). Some studies have also demonstrated that NSAIDs have affinity with PPARs; indeed, indomethacin is used in combination with other compounds for adipocyte differentiation in vitro. A study by Lehmann et al. showed that indomethacin, and other NSAIDs like ibuprofen and flufenamic acid, bind to PPAR-γ and stimulate adipogenic differentiation in vitro [31]. However, the nature of NSAID/PPAR-γ interaction has not been fully elucidated. In fact, according to different in vitro research, indomethacin-dependent activation of PPAR-γ changes according to the experimental concentration [31]. For these reasons, the present review will be mainly focused on well-established PPAR agonists, TZDs.

TZDs, which include troglitazone, rosiglitazone, and pioglitazone, are insulin-sensitizing drugs, originally identified as compounds able to regulate insulin and glucose levels. Currently, rosiglitazone and pioglitazone are the only clinically used drugs for the treatment of type 2 diabetes mellitus [32], whereas troglitazone has been interrupted for liver toxicity. TZDs increase tissue sensitivity to insulin and glucose uptake in insulin-sensitive tissues, adipose tissue, liver, and skeletal muscle by acting on various mechanisms [32,33]. PPAR-γ activation by TZDs stimulates adipocyte differentiation, increasing the number of small adipocytes that are able to store free fatty acids (FFA), thus lowering the circulating levels of FFA. Further, TZDs induce the fatty acid translocase, a glycoprotein addressed to FFA transport to adipose tissue [34]. These actions limit the amount of lipid storage in liver and muscle and allow the distribution of fat from visceral to subcutaneous tissues. TZDs also manage the glucose content, by inhibiting the apoptosis of pancreatic β-cells and improving insulin secretion [32,33]. PPAR-γ activation by TZDs also determines the reduction of pro-inflammatory genes like TNF-α and IL-6 and inhibits the expression of NF-κB. TZDs also exert antithrombotic and antiatherogenic effects, acting on both endothelial cells and SMCs. In this regard, TZDs stimulate endothelial cells to secrete vasoactive molecules, thus preserving the endothelial function [35]. In addition, TZDs inhibit SMC proliferation and migration [36]. Our research group recently tested the in vitro effects of pioglitazone on MSCs obtained from human AAAs, observing that the PPAR-γ induction by drug administration correlated with a significant down-regulation of matrix metalloproteinase-9 (MMP-9) predominantly involved in pathological vascular remodeling during aneurysm and atherosclerosis pathogenesis [37].

Considering the high impact of PPAR-γ activation on the inflammatory process, the use of PPAR-γ agonists has been proposed among the possible therapeutic compounds able to treat the cytokine storm that typically occurs during severe viral influenza. In this regard, a 2009 study by Aldridge et al. demonstrated that pioglitazone administration to mice reduced the amount of dendritic cell recruitment in infected lung and expanded the rate of CD8+ T cells, resulting in reduced morbidity and mortality due to highly pathogenic (HP) influenza virus A [38]. A 2010 study by the same group further supported the therapeutic use of TZDs, showing the efficacy of both rosiglitazone and pioglitazone on reducing the inflammatory process induced by the cytokine storm in a mouse model of H1N1 influenza A virus [39].

The recent emergency due to the worldwide spreading of COVID-19 calls for an urgent search of novel and effective therapeutic strategies aimed at preventing virus replication and the structural and functional assembly or the synthesis of viral RNA. A computational study by Wu et al. [40] identified different classes of compounds that potentially affect coronavirus infection. Among these, pioglitazone was included in the group of drugs inhibitor of the viral papain-like protease (PLpro), essential for virus replication and host infection [41,42,43,44].

**Figure 2:**
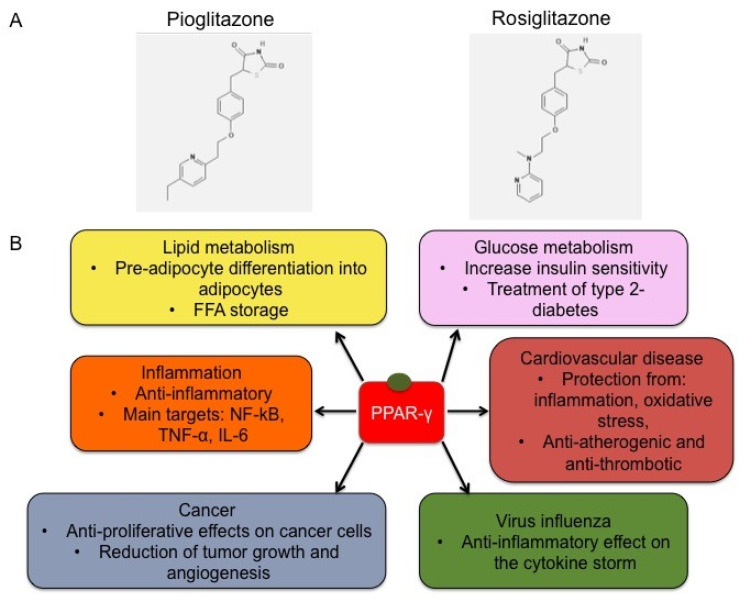
Chemical structure of representative TZDs and functions. (**A**) Chemical structure of pioglitazone (CID = 4829) and rosiglitazone (CID = 77999) [45]. (**B**) Schematic representation of TZD effects. The binding of TZDs, like pioglitazone and rosiglitazone, to PPAR-γ induces its activation, triggering the modulation of target gene expression and a broad range of mechanisms, including lipid metabolism, regulation of glucose levels, inflammatory processes, cell proliferation and migration, cardiovascular diseases and vascular remodeling, and cytokine storm occurrence under virus infections.

A summary of the broad spectrum of therapeutic effects exploited by the synthetic PPAR-γ agonists is graphically summarized in Figure 2.

**Figure 3:**
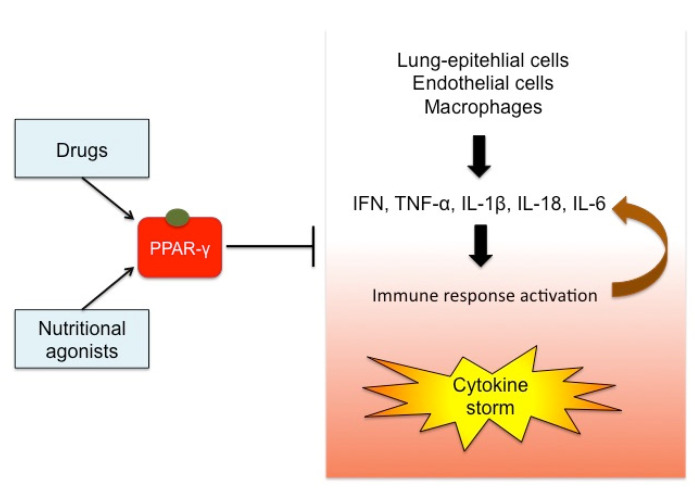
Effects of PPAR-γ activation on cytokine storm. The stimulation of PPAR-γ by drugs or foods can exert a regulatory role on the cytokine storm typical of virus infections. Pro-inflammatory cytokines (e.g., IFN, TNF-α, IL-1β) are released by lung-epithelial cells, endothelial cells, and immune cells, inducing the immune system response. This mechanism recruits chemokines and other cytokines, in a vicious circle out of control. PPAR-γ acts on the transcription of the upstream inflammatory genes, thus preventing the cytokine over-production and becoming an attractive target for immunomodulatory therapies in the case of viral infections.

Based on these considerations, PPAR-γ could represent a promising target for treating viral influenza associated with the inflammatory circuit that occurs during the cytokine storm. This mechanism is depicted in Figure 3.

The wide clinical implications of targeting PPAR-γ encouraged the study and development of a novel class of PPAR-γ agonists, like dual-PPAR and pan-PPAR agonists, called glitazars [46]. Among these, the PPARα/γ dual agonists have been proposed for treatment of type 2 diabetes and dyslipidemia, demonstrating also anti-inflammatory and anti-coagulant effects [10]. However, some critical points like the safety, effectiveness, and potential side effects of these novel drugs are still under investigation. Therefore, exploring the main activation mechanisms of novel PPAR ligands as well as their pharmacodynamics represents a promising research area, paving the way to more potent and effective therapeutic strategies in many disease fields. A comprehensive overview of PPAR modulators and their current clinical status is reviewed elsewhere [46].

## 3. Nutritional Ligands of PPAR-γ

A series of natural PPAR-γ ligands that can be found in food are listed and discussed below.

### 3.1. Sea Food and Fish Oil

Polyunsaturated fatty acids are related to the modulation of inflammation; in particular, they are responsible for a decrease of the production of pro-inflammatory cytokines through activation of PPAR-γ [30]. Docosahexaenoic acid (DHA) and eicosapentaenoic acid (EPA) are contained in sea food and fish oil, and they are involved in the inhibition of various aspects of inflammation, such as leucocytes chemotaxis, adhesion molecule expression, and eicosanoid production. Their interaction with PPAR-γ leads to the inhibition of NF-κB, a key transcription of pro-inflammatory cytokine production. In particular, they inhibit the translocation of NF-κB into the nucleus, with consequent down-regulation of its target genes and, therefore, of the expression of inflammatory proteins [47].

### 3.2. Turmeric

Turmeric is obtained from Curcuma longa, a plant cultivated in Asia. The rhizome is the most useful and used part of the plant; it is generally consumed as a food ingredient, but in the past years it has also been used as a treatment for wound healing and inflammatory disorders [48]. Curcumin, the phytochemical component of turmeric, is able to regulate the expression of numerous transcription factors, cytokines, adhesion molecules, and enzymes related to inflammation [49]. The anti-inflammatory effect of curcumin is mediated by the induction of PPAR-γ up-regulation, which consequently leads to the inhibition of the expression of TNF-α, a cytokine involved in inflammation [48]. The mechanism by which curcumin stimulates PPAR-γ expression has not yet been clarified. Nevertheless, curcumin induces an anti-inflammatory effect through the up-regulation of PPAR-γ, which leads to the inhibition of NF-κB, a pro-inflammatory mediator [50].

### 3.3. Thyme and Oregano

Thyme and oregano are typical plants of the Mediterranean area that have a culinary use as aromatic herbs. They contain carvacrol, a monoterpenic phenol, used as a food flavoring and as a fragrance in cosmetics. In vitro and in vivo studies show that carvacrol has antioxidant, antibacterial, and anti-inflammatory properties [51]. Its anti-inflammatory property is associated with its role in the inhibition of COX-2; in fact, carvacrol plays an agonistic role on PPAR-γ, which induces the inhibition of the activity of the COX-2 promoter. COX-2 is the inducible form of COX, an enzyme that converts arachidonic acid into prostaglandins, which mediate and promote the induction of inflammation [52].

### 3.4. Hot Pepper

Hot pepper is a member of the Solanaceae family; it is the most widespread spice in the world, and it is generally used as an ingredient in cooking. It contains capsaicin, the most present compound in hot pepper, which, together with other capsaicinoids, is responsible for the spiciness. Capsaicinoids have antifungal and antibacterial properties and have been used to treat arthritis [53]. Capsaicin has anti-inflammatory properties. A study has shown that it inhibits the lipopolysaccharide (LPS)-induced production of TNF-α, a pro-inflammatory cytokine, by activating PPAR-γ. It can therefore represent a naturally occurring ligand for PPAR-γ, stimulating anti-inflammatory mechanisms [54].

### 3.5. Rosemary and Sage

Rosemary and sage are herbs that are mainly found in the Mediterranean area; they are used in the kitchen as aromatic plants, but in the past, they have been also used for their antiseptic, antioxidant, and anti-inflammatory properties. They contain dipertenoids, such as carnosic acid and carnosol, which have been shown to have anti-inflammatory characteristics. Indeed, carnosic acid and carnosol interfere with the key pathways of inflammation, inhibiting the activation of NF-κB and the production of COX-2, eicosanoids, IL-1β, and TNF-α, all inflammatory products [55]. Furthermore, carnosic acid and carnosol have been shown to have agonistic effects, thus activating PPAR-γ. This may represent a basis for the development of PPAR-γ activators with anti-inflammatory activity [56].

### 3.6. Pomegranate

Pomegranate is a fruit grown mainly in Southeast Asia, the Mediterranean region, and in the USA. It has been used for thousands of years as a medicinal fruit for its potential beneficial properties; in fact, the various parts of the fruit seem to contain antimicrobial, antifungal, antioxidant, and anti-inflammatory compounds. In particular, pomegranate seed oil contains punicic acid, a conjugated alpha-linoleic acid with anti-inflammatory and immunomodulatory properties [57]. Punicic acid inhibits the expression of pro-inflammatory cytokines, such as IL-6, IL-8, IL-12, and TNF-α, through PPAR-γ modulation, which inhibits the expression of the NF-κB pathway and suppresses expression of pro-inflammatory M1 macrophages while promoting the differentiation of anti-inflammatory M2 macrophages [58].

### 3.7. Lemongrass

Lemongrass is a perennial herb used mainly for the extraction of its oil, which is used as an aroma in the kitchen and as a fragrance in cosmetics. Lemongrass has been used for years, especially in Southeast Asia, for its analgesic and anti-inflammatory properties [59]. Lemongrass oil contains citral, which gives it the distinctive aroma and perfume and is a mixture of aldehydes that have the same molecular formula but different structure, the trans isomer geranial and the cis isomer neral. Citral activates PPAR-γ, which inhibits the expression of pro-inflammatory cytokines, such as IL-1β and IL-6, and suppresses the activity of the COX-2 promoter [60].

The chemical structure of the natural ligands of PPAR-γ has been collected from PubChem [45] and is reported in Table 1.

A further and detailed selection of bioactive compounds able to activate PPAR-γ (i.e., flavonoids, neolignans, stillbenes, and armofrutins) is reviewed elsewhere [61].

Herbs and spices, commonly intended for culinary purposes, have been used for centuries for the treatment of various diseases, especially in the past, and still represent the source for identifying compounds used for development of new synthetic drugs. The effects of these compounds are usually dose-dependent; for example, curcumin needs a very high dose to produce a medicinal effect in humans (>3.6 g/day) and, additionally, it has a low bioavailability, which limits its use [62]. This does not permit a pharmacological effect from the administration of the plant in its entirety. The European Food Safety Authority (EFSA), in fact, does not report any indication regarding the doses and concentrations that exert a pharmacological effect, since it does not recognize the clinical efficacy of the anti-inflammatory properties of these foods [63]. On the other hand, some studies have shown that the consumption of some types of food contribute to the treatment of human disorders [64]. Hermsdorff et al. investigated the potential relationship between food and inflammation, observing that adults with a high consumption of fruit and vegetables showed lower expression levels of inflammation-related genes (IL-6, TNF-α, NFκB1) [65]. This suggests a correlation between food and its potential beneficial effect in reducing inflammation. The mechanism by which fruits, vegetables, and herbs contribute to decreasing inflammation levels is not yet clear; in fact, additional studies will be needed to explore and clarify the mode of action of phytochemicals.

Certainly, in a context of clinical severity, one cannot expect a therapeutic efficacy of these foods; however, they could represent an integrative method to enhance the immune system if used as dietary supplements or studied for the identification of new therapeutic PPAR-γ agonists.

## 4. Conclusions

Currently, the new coronavirus infection (COVID-19) represents a serious worldwide health problem due to its diffusion and the rate of hospitalization and death, which put national health systems under critical stress. The severe progression of this condition has been related to the cytokine storm, an overwhelming cytokine production associated to some viral threats that is characterized by an altered host inflammatory response. In the absence of an effective vaccine or antiviral therapy, immunomodulatory therapy could be an effective approach aimed at regulating cytokine levels.

In this context, the activation of PPAR-γ through synthetic and nutritional compounds could represent an effective therapeutic strategy to contrast this cytokine storm and to prevent the insidious inflammatory effects following coronavirus infections. PPAR-γ, a transcription factor member belonging to the PPAR family and known as a regulator of lipid and glucose metabolism, is involved in other regulatory activities such as adipocytes differentiation, cell proliferation, the immune cell system, and inflammatory response. Although PPAR-γ synthetic agonists, e.g., pioglitazone, are clinically employed as drugs for the treatment of type 2 diabetes mellitus, these anti-diabetic drugs have anti-inflammatory properties making them promising candidates to treat inflammation in severe viral disease suggesting possible applications as modulators of host inflammatory and immune responses due to viral infections.

Moreover, nutritional ligands able to activate PPAR-γ have been identified in a wide range of natural products, including sea food and fish oil, pomegranate, and herbs and spices (curcuma, thyme, oregano, hot pepper, rosemary, sage, lemongrass), commonly used in cooking. These nutritional PPAR-γ agonists exert inhibitory effects on pro-inflammatory cytokines and promote immune cell differentiation into anti-inflammatory phenotypes. These foods could represent a natural anti-inflammatory defense useful to strengthen individual health. The promotion of a more conscious lifestyle characterized by a diet rich in foods, herbs, and spices capable of activating PPAR-γ could be a valid option to strengthen the immune system via natural methods in order to prevent cytokine storm occurrence in the case of infections due to coronavirus.

## Figures and Tables

**Table 1:** Nutritional agonists of PPAR-γ. The table summarizes the main nutrients activator of PPAR-γ and their chemical structures.

PPAR-γ Agonists	Structure	Food
Docosahexaenoic acid (DHA)	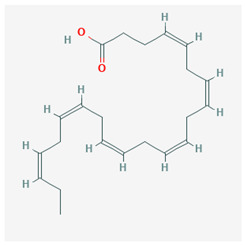 CID = 445,580	Sea food and fish oil
Eicosapentaenoic acid (EPA)	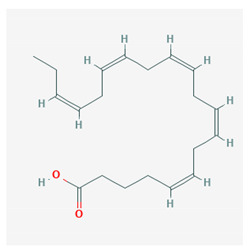 CID = 446,284	Sea food and fish oil
Curcumin	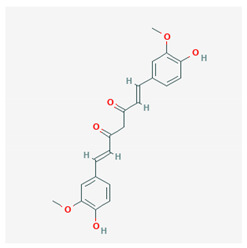 CID = 969,516	Turmeric
Carvacrol	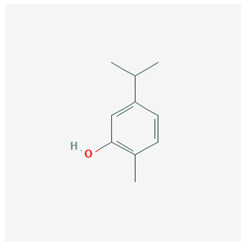 CID = 10,364	Thyme and oregano
Capsaicin	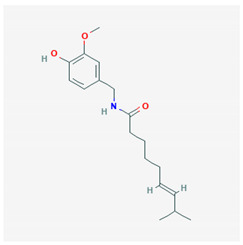 CID = 1,548,943	Hot pepper
Carnosic acid	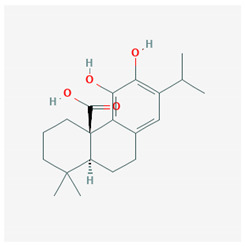 CID = 65,126	Rosemary and sage
Carnosol	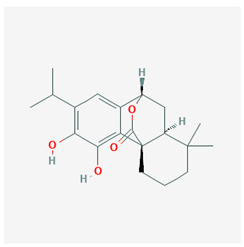 CID = 442,009	Rosemary and sage
Punicic acid	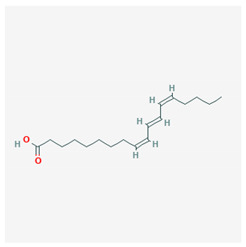 CID = 5,281,126	Pomegranate seed oil
Citral	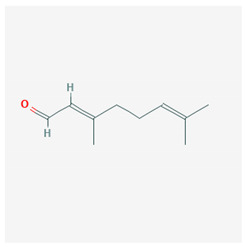 Geranial CID = 638,011 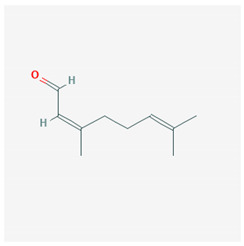 Neral CID = 643,779	Lemongrass
